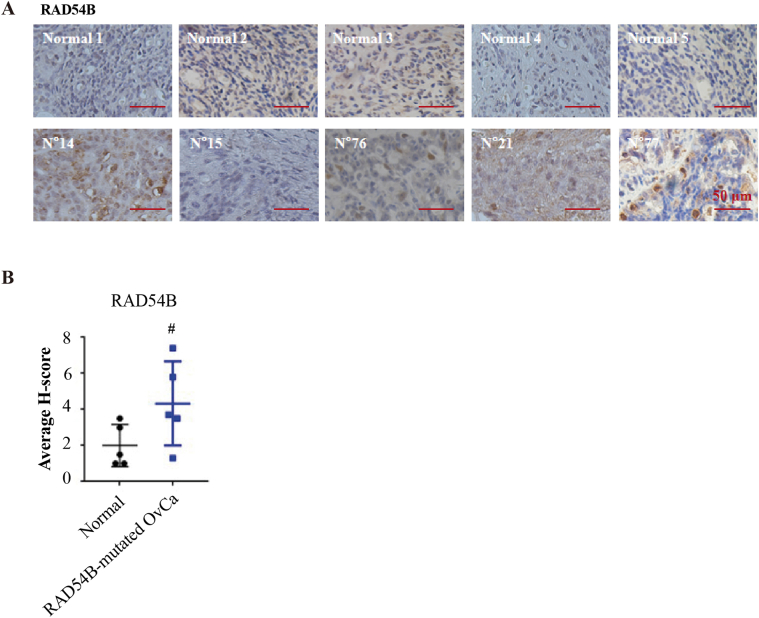# RAD54B mutations enhance the sensitivity of ovarian cancer cells to poly(ADP-ribose) polymerase (PARP) inhibitors

**DOI:** 10.1016/j.jbc.2022.102354

**Published:** 2022-08-09

**Authors:** Peng Liu, Chunxiu Lin, Lanlan Liu, Ziwen Lu, Zhigang Tu, Hanqing Liu

**Affiliations:** 1 School of Life Sciences, Jiangsu University, Zhenjiang, Jiangsu, China; 2 School of Pharmacy, Jiangsu University, Zhenjiang, Jiangsu, China

**Keywords:** ovarian cancer, PARP inhibitors, olaparib, RAD54B, whole-exome sequencing, homologous recombination, DSB, double-strand break, FA, Fanconi anemia, FDA, Food and Drug Administration, FFPE, formalin-fixed paraffin-embedded, GATK, Genome Analysis ToolKit, HR, homologous recombination, HRD, HR deficiency, IHC, immunohistochemistry, OvCa, ovarian cancer, PARP, poly(ADP-ribose) polymerase, PARPi, poly(ADP-ribose) polymerase inhibitor, WES, whole-exome sequencing

## Abstract

Synthetic lethal targeting of homologous recombination (HR)–deficient ovarian cancers (OvCas) with poly(ADP-ribose) polymerase inhibitors (PARPis) has attracted considerable attention. Olaparib was the first PARPi approved by the Food and Drug Administration, offering significant clinical benefits in BRCA1/2-deficient OvCas. However, only approximately 20% of OvCa patients harbor *BRCA1/2* mutations. Given the shared roles that BRCA1/2 have with other HR regulators, alterations in HR genes may also contribute to “BRCAness profiles” in OvCas. RAD54B has been considered a key player in HR repair, although its roles and therapeutic potential in cancers need further investigation. Here, we identified 22 frequently mutated HR genes by whole-exome sequencing of OvCa tissues from 82 patients. To our surprise, 7.3% of patients were found to harbor mutations of *RAD54B*, the third-highest mutated gene among patients. We determined that *RAD54B*-mutated tumor tissues harbored more DNA double-strand breaks than normal tissues. Additionally, we found that *RAD54B* knockdown inhibited HR repair, enhanced sensitivities of OvCa cells with increased DNA double-strand breaks to olaparib, and induced apoptosis. Enhanced inhibitory effects of olaparib on the growth of ES2 xenograft tumors were further demonstrated by *RAD54B* knockdown. Finally, we show that restoration with wildtype RAD54B rather than RAD54B^N593S^ and RAD54B^H219Y^, identified in patients, abolished the effects of *RAD54B* knockdown, indicating these *RAD54B* mutants probably malfunctioned in HR repair. Our investigations may offer insight into the contributions of *RAD54B* mutations to synthetic lethality with olaparib treatment in OvCas, enrich the gene list for “HR deficiency scoring,” and expand the applications of PARPis.

Ovarian cancer (OvCa) is one of the most common gynecological malignancies worldwide with the highest mortality rate (1). Despite advances in therapies over the past 2 decades, 5-year survival rate is only about 50% (2). OvCa often manifests with frequent genetic alterations in DNA repair–related genes (3, 4, 5, 6). In particular, the defects in homologous recombination (HR) genes, such as *BRCA1/2*, *ATM*, *ATR*, *RAD51*, and Fanconi anemia (FA) genes, account for approximately 36% of all OvCas and half of high-grade OvCas (4, 5, 6, 7). In this scenario, growing attention has been paid to therapeutic strategies targeting DNA repair against OvCas.

Synthetic lethality is one such anticancer therapeutic strategy, which takes advantages of lethal combination of two independently viable mutations (8). In OvCa therapy, this strategy exploits specific DNA repair–related genetic lesions in cells by targeting synthetic lethal partner genes. The synthetic lethal therapy can in principle result in selective OvCa cell death, while avoiding normal cells harmed. In HR-defective OvCas, poly(ADP-ribose) polymerases (PARPs) are considered as attractive synthetic lethal targets because of their crucial roles in DNA single-strand break repair (9, 10). PARP inhibitors (PARPis) can inhibit PARP-dependent single-strand break repair, leading to accumulation of fatal double-strand breaks (DSBs) that causes synthetic lethality in HR-defective OvCa cells. The first PARPi approved by Food and Drug Administration (FDA) was olaparib (11, 12, 13). Evidence from phase I, II, and III studies demonstrated high clinical efficacy and excellent tolerability of olaparib therapy in *BRCA1/2*-mutated OvCa patients (14, 15, 16, 17, 18, 19). However, it is noteworthy that only approximately 20% of OvCa patients have *BRCA1/2* mutations (4, 6, 13). Defects in other HR-related genes, such as *RAD51*, *RAD54*, and *PTEN* genes, may also contribute to a “BRCAness profile” (4, 20, 21).

In the present study, we carried out a series of whole-exome sequencing (WES) on DNAs extracted from OvCa tissues of patients and identified the most frequently mutated genes in HR pathways. These highly mutated HR genes include *BRCA1*, *BRCA2*, *RAD54B*, *FANCM*, *ATR*, *ATM*, *RAD51*, and *PALB2*, and others. The regulatory mechanisms of these genes in DNA repair and the synthetic lethal effects when PARPi treatments were applied in various cancers with defective HR genes have been studied (22, 23, 24, 25, 26, 27, 28, 29, 30). RAD54B-deficient colorectal cancer cells were reported to increase accumulation of DNA DSBs and apoptosis under olaparib treatment (30). Its therapeutic value in OvCa remains unclear.

RAD54B was first isolated as a human RAD54 homolog in 1999 (31). Given that RAD54B interacts with RAD51 and DMC1 to enhance the D-loop formation and DNA-strand exchange during HR, this protein was suggested to be critical in DNA repair and harbored with tumor suppressor–like properties (32, 33, 34, 35). RAD54B was also found to form nuclear foci with hRAD54, hRAD51, and BRCA1 (36, 37). Although more and more researchers have paid attention to the roles of RAD54B in anticancer therapy (38, 39), the roles and molecular mechanisms of malfunctions of RAD54B in OvCa need further investigations. Here, we focused on RAD54B to explore whether RAD54B defects rendered OvCa cells more sensitive to a PARPi olaparib.

## Results

### WES identified frequent mutations of HR-related genes in OvCa tissues

**Figure 1:**
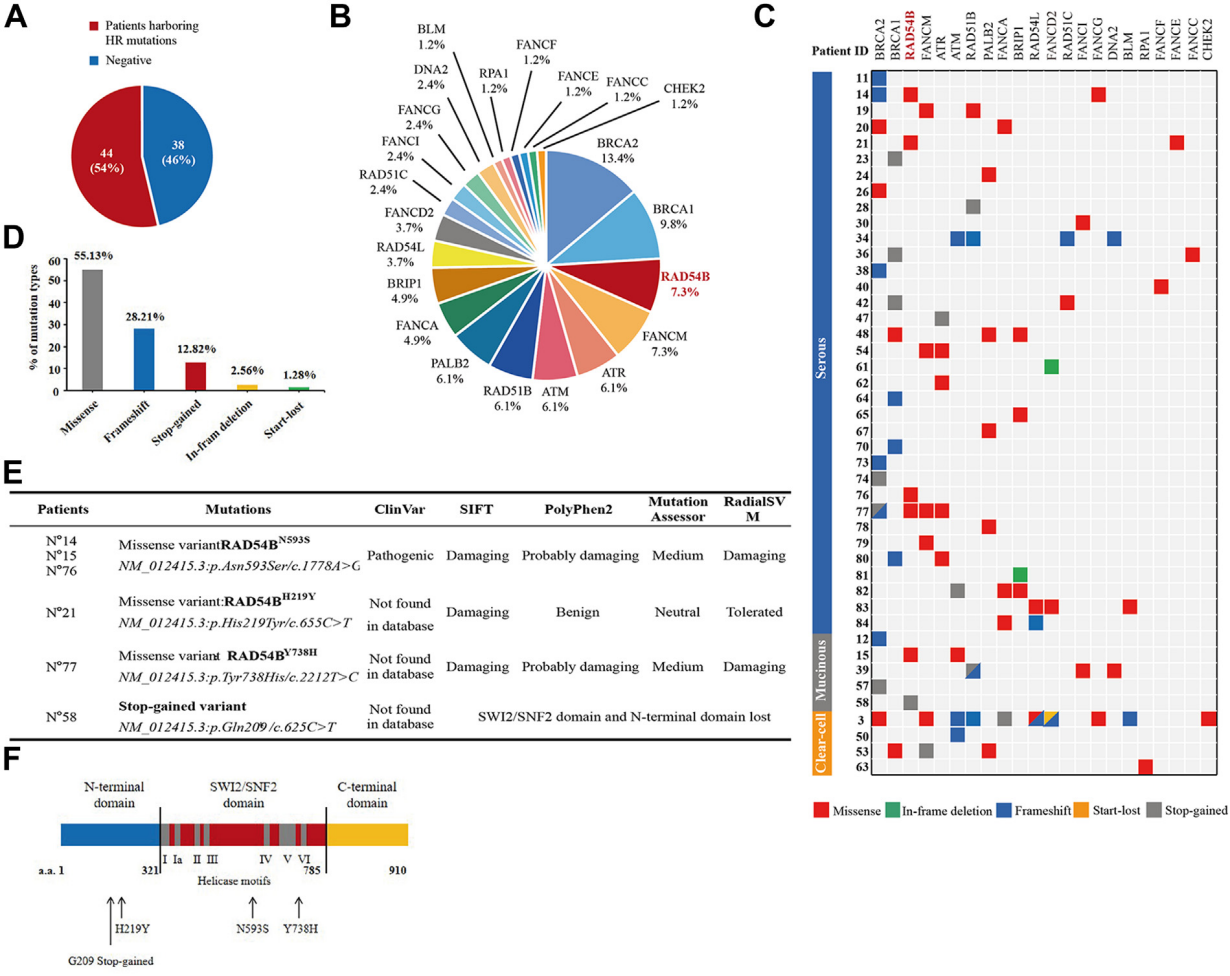
**Whole-exome sequencing (WES) identified frequent mutations of HR-related genes in ovarian cancer (OvCa) tissues and pathogenicity prediction of *RAD54B* mutations.***A*, in the total 82 patients, OvCa tissues of 44 patients (54%) harbored mutations in HR-related genes. *B*, percentages of patients harboring mutations in each HR-related gene. *C*, WES-identified HR mutations in OvCa tissue specimens. Mutations were shown in the *central box* with colors indicating mutation types. The *left bars* represented the histological subtypes of the OvCa cases. *D*, percentages of missense, frameshift, stop-gained, in-frame deletion, and start-lost variants in all HR variants identified. *E*, pathological relevance of the *RAD54B* mutations predicted by SIFT, PolyPhen2, Mutation assessor, and RadialSVM platforms. *F*, the positions of mutations in RAD54B protein identified by WES. HR, homologous recombination.

Started from the paraffin-embedded tumor tissues of 84 OvCa patients, the WES was successfully performed on extracted DNAs from 82 patients. The major clinical and pathological characteristics of the successfully sequenced patients are listed in Table S1. The genomic DNAs of all these patients were extracted and quality controlled. Sequencing was conducted using the next-generation sequencing BGISEQ-500 platform, achieving a mean clean read rate at 99.02 ± 0.59%. The average Q20 and Q30 of each clean read were 97.84 ± 0.60% and 91.21 ± 1.45%, respectively. The subsequent alignment, mapping, and variant calling were carried out using Burrows-Wheeler Aligner/Genome Analysis ToolKit (GATK)/HaplotypeCaller/SnpEff softwares (Broad Institute). In all 82 cases, a total of 9,161,917 single nucleotide variants and 1,629,471 insertions–deletions were identified, implying a mean rate of 6.72 ± 1.45 mutations per megabase. Excluding the mutations with frequencies higher than 1% in 1000 human genome project database, NHLBI-ESP6500 European American database, and NHLBI-ESP6500 African American database, our analyses identified 22 mutated HR-related genes (*BRCA1*, *BRCA2*, *RAD51B*, *FANCM*, *ATR*, *ATM*, *RAD54L*, *RAD54B*, *PALB2*, *FANCD2*, *FANCA*, *BRIP1*, *RAD51C*, *FANCI*, *FANCG*, *DNA2*, *BLM*, *RPA1*, *FANCF*, *FANCE*, *FANCC*, and *CHEK2*) with 78 potential pathogenic variants from 44 patients by using SIFT (http://sift.jcvi.org/)/PolyPhen2 (http://genetics.bwh.harvard.edu/pph2/)/Mutation assessor (http://mutationassessor.org/)/Radial SVM software platforms (Fig. 1, *A*–*C* and Table S2). The 78 HR-related variants included 43 missense variants (55.13%), 22 frameshift variants (28.21%), 10 stop-gained variants (12.82%), 2 in-frame deletion variants (2.56%), and 1 start-lost variant (1.28%) (Fig. 1*D*). The clinical significance and status of all these 78 variants in cancers were characterized in COSMIC and ClinVar databases, and they were also analyzed and predicted by using SIFT/PolyPhen2/Mutation assessor/Radial SVM software platforms (Table S2).

Among the total 82 OvCa patients, 23.2% of patients had *BRCA1* or *BRCA2* mutations (Fig. 1*B*), and roles of *BRCA1/2* in outcomes of PARPi-treated OvCas have already been extensively studied (14, 40, 41). In addition, 24.3% of patients had mutations in FA proteins, including FANCM (7.3%), FANCA (4.9%), FANCD2 (3.7%), FANCI (2.4%), FANCG (2.4%), and others (Fig. 1*B*). In view of the burgeoning evidence that FA/BRCA pathways regulated repair of DNA interstrand crosslinks through HR, it seemed that the FA defects contributed to the synthetic lethality in cancers treated with interstrand crosslink–inducing drugs (28, 29, 42, 43). Other highly mutated HR-related genes, including *RAD51B* (mutation frequency in patients, 6.1%), *ATR* (6.1%), *ATM* (6.1%), *PALB2* (6.1%), and *BRIP1* (4.9%) (Fig. 1, *B*), have been reported to be capable of affecting the sensitivity of various cancer cells to PARPis (22, 23, 24, 25, 26, 27).

Interestingly, 7.3% of patients had *RAD54B* mutations (Fig. 1*B*). Four different *RAD54B* mutations were identified in six OvCa patients (Fig. 1*C*). Earlier studies have shown that *RAD54B* is an important player in HR pathway (31). However, its importance in anticancer therapy needs further investigations. In fact, alterations of *RAD54B* have been detected in several cancers, including lung adenocarcinoma, breast cancer, and hepatoma (44, 45, 46, 47). *RAD54B* silencing often leads to reduced cell proliferation and enhanced apoptosis (44, 47). Moreover, in colorectal cancer cells, inactivation of *RAD54B* was reported to increase DSBs and apoptosis (37, 48, 49). Here, since 7.3% of OvCa patients harbored *RAD54B* mutations, the third highest mutated gene among these patients, we therefore focused on *RAD54B* to gain further insight into its therapeutic values in OvCas.

### Pathogenicity prediction of *RAD54B* mutations

To predict the importance of *RAD54B* mutations in OvCa therapy, we first employed SIFT/PolyPhen2/Mutation assessor/Radial SVM software platforms. The clinical relevance of these variants was further analyzed and identified in COSMIC and ClinVar databases. The aforementioned WES analyses identified four *RAD54B* mutations, including N593S, H219Y, Y738H, and one stop-gained variant, in OvCa tissues of six patients (Fig. 1*E*). N593S was located at the conserved region between helicase motifs III and IV in SNF2 domain of RAD54B protein (Fig. 1*F*) (31). This mutation was observed in human primary lymphoma and colon cancer (31) and also found in acute myeloid leukemia (legacy mutation ID: COSM4416566) recorded in ClinVar database. To the best of our knowledge, the other two *RAD54B* missense mutations, H219Y and Y738H, were identified the first time in cancer cells. These two *RAD54B* missense mutations were not included in National Center for Biotechnology Information ClinVar or COSMIC databases. The RAD54B variants H219Y and Y738H were predicted to be damaging by SIFT platform and probably damaging by PolyPhen2 platform (Fig. 1*E*). In addition, a stop-gained mutation (CAG to TAG) at codon 209 was also identified. This stop-gained mutation was located at the N-terminal domain, which can lead to the loss of SNF2 domain and C-terminal domain in RAD54B protein (Fig. 1*F*). Loss of SNF2 domain can thus result in the disruption of ATPase activity, which was required for the translocation of RAD54B along duplex DNA and DNA double-helix opening (50). Collectively, these bioinformatic predictions suggested that these four *RAD54B* mutations can probably affect HR repair functions of RAD54B.

### *RAD54B*-mutated tumors harbored more DNA DSBs

**Figure 2:**
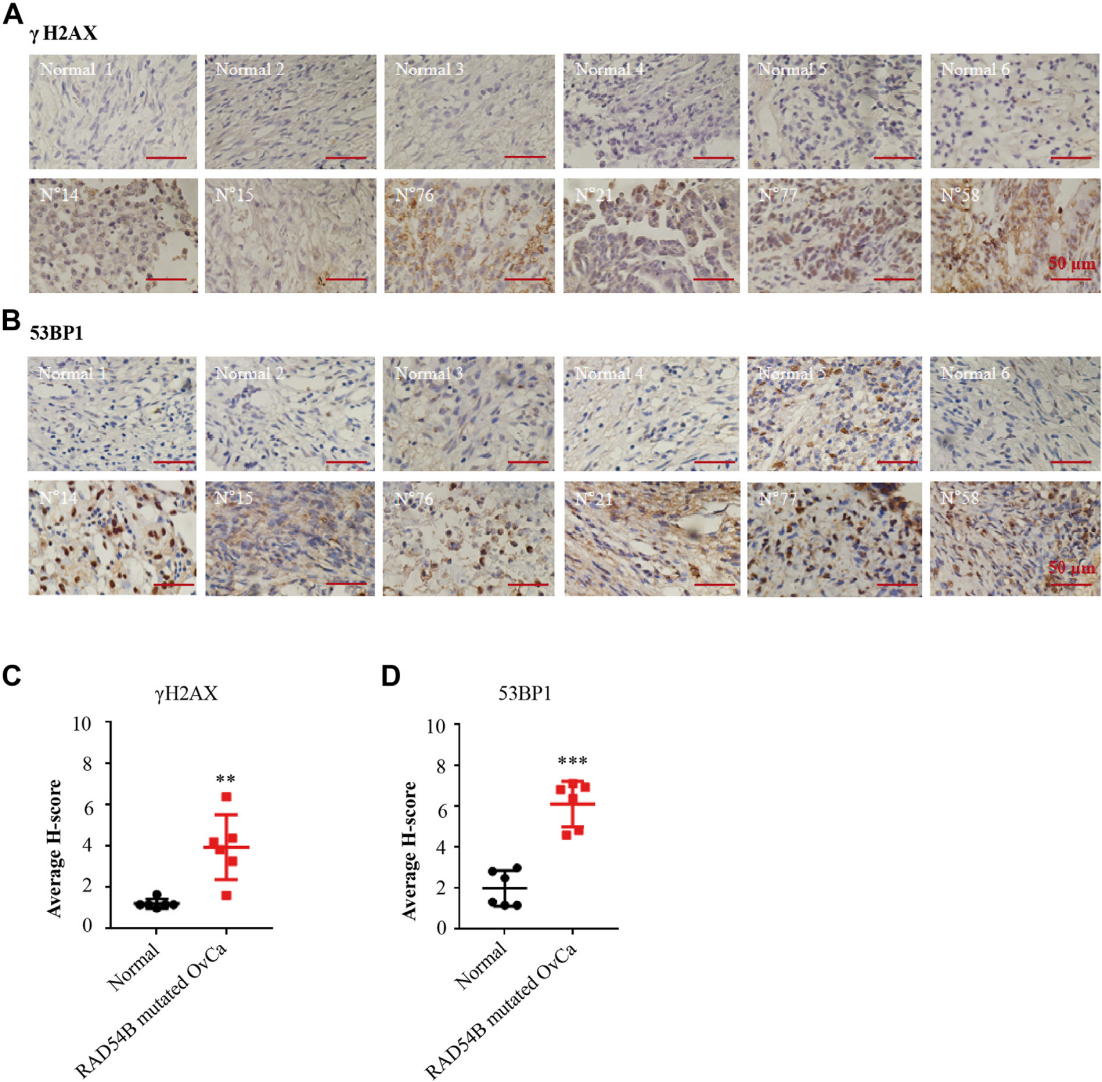
**Accumulation of DSBs in *RAD54B*-mutated ovarian cancer (OvCa) tissues.***A* and *B*, representative images of IHC showing γH2AX and 53BP1 staining in OvCa tissues from the six patients harboring *RAD54B* mutations and in normal ovarian epithelial tissues. The scale bar represents 50 μm. *C* and *D*, statistical analyses of the average H-scores of γH2AX and 53BP1 staining in six *RAD54B*-mutated OvCa samples and in six normal ovarian epithelial tissues. Mean ± SD, n = 6. ∗∗*p* < 0.01 and ∗∗∗*p* < 0.001. DSB, double-strand break; IHC, immunohistochemistry.

As the next step, immunohistochemistry (IHC) analyses were performed in OvCa tissues of the six patients harboring *RAD54B* mutations (patient ID nos.: 14, 15, 21, 58, 76, and 77). Two classical biomarkers for DNA DSBs, γ-H2AX and 53BP1 (51, 52, 53, 54, 55), were used to assess the amounts of DSBs in *RAD54B*-mutated OvCa specimens. As expected, the H-scores of γ-H2AX and 53BP1 were significantly higher in *RAD54B*-mutated OvCa tissues (n = 6), compared with those in normal ovarian epithelial tissues (n = 6) (*p* = 0.0017 for γ-H2AX and *p* = 0.0003 for 53BP1, respectively) (Fig. 2, *A*–*D*). These results demonstrated that there were significantly more DSBs in *RAD54B*-mutated OvCa tissues when compared with the normal ovarian epithelial tissues.

Considering that RAD54B is an important protein involved in HR repair and taking into account the aforementioned prediction results *via* bioinformatics, we speculated that *RAD54B* mutations may affect its intracellular functions, thereby impairing DSB repair. To test this hypothesis, we conducted the following *in vitro* experiments.

### *RAD54B* knockdown inhibited HR repair in OVCAR8 cells

**Figure 3:**
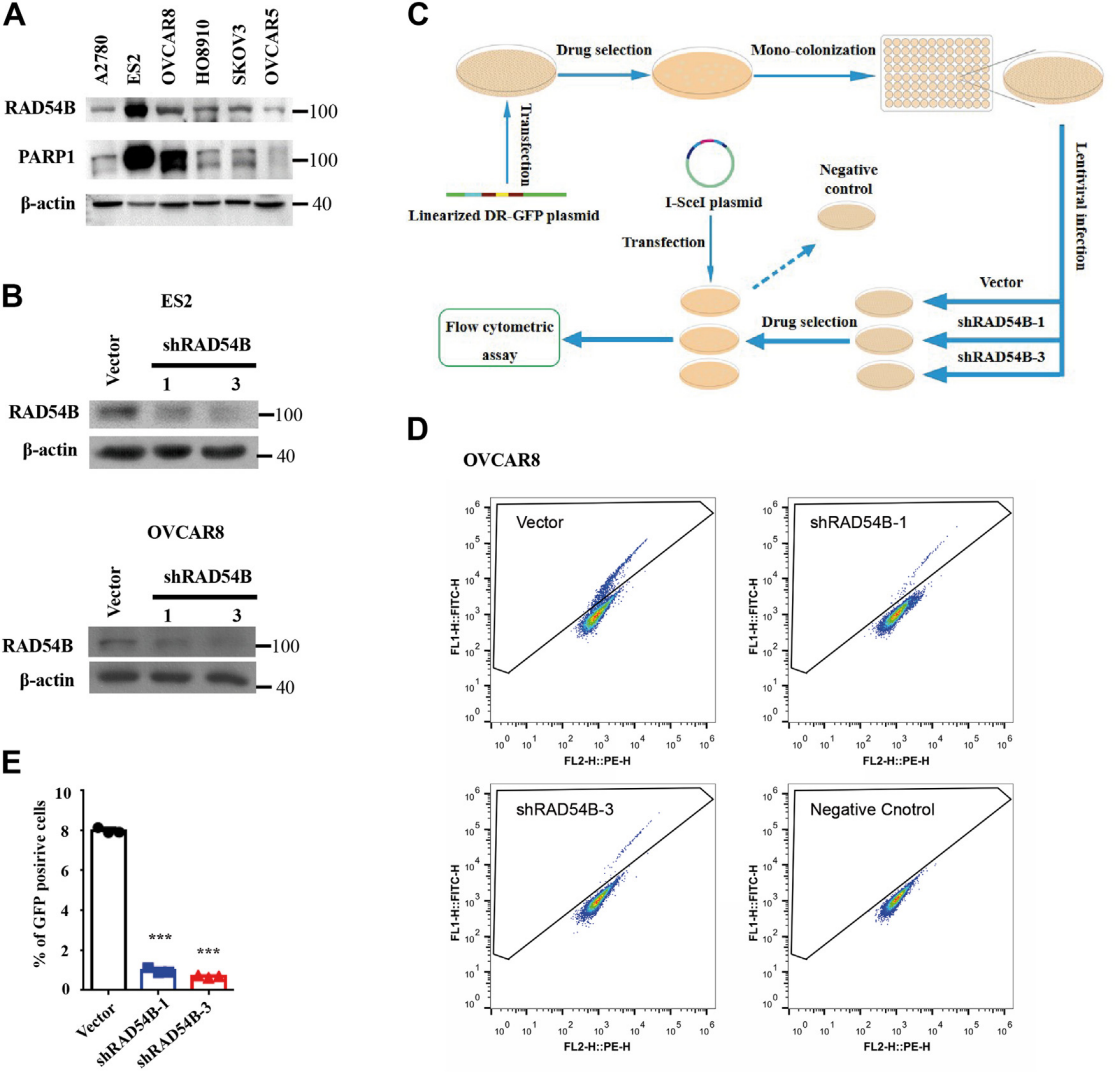
***RAD54B* knockdown inhibits HR repair in ovarian cancer (OvCa) cells**. *A*, protein levels of RAD54B and PARP in six OvCa cell lines. *B*, *RAD54B* knockdown in OvCa cell lines with shRAD54B-1 and shRAD54B-3. *C*, schematic diagram depicting the DR-GFP assay. *D*, representative images of flow cytometry showing the proportions of GFP-positive cells in DR-GFP assays in OVCAR8 cells. *E*, statistical analysis of the percentages of GFP-positive cells in *RAD54B* knockdowned OVCAR8 cells and OVCAR8 cells with empty vectors. Mean ± SD, n = 3. ∗∗∗*p* < 0.001. HR, homologous recombination; PARP, poly(ADP-ribose) polymerase.

To facilitate subsequent *in vitro* experiments, we first screened six OvCa cell lines preserved in our laboratory. Two cell lines, ES2 and OVCAR8, were selected for their abundant protein levels of RAD54B and PARP1 (Fig. 3*A*). To simulate the functional defects of RAD54B, its protein levels were knockdowned by using two different shRNAs (Fig. 3*B*). To test the aforementioned hypothesis, we used the DR-GFP assay, a commonly used method, to test intracellular HR repair abilities (56, 57, 58) in the following experiments (Fig. 3*C*). OVCAR8 cells were stably transfected with DR-GFP, and the assays were accomplished. The HR repair abilities of different groups were analyzed using flow cytometry, and the vector group without I-SceI transfection was used as the negative control. As shown in Figure 3, *D* and *E*, when I-SceI enzyme was transiently expressed, a portion of cells in the vector group gave GFP signals after HR, whereas almost none signals for the negative control group without I-SceI transfection. More importantly, when compared with the vector group, two groups with shRAD54B knockdown exhibited dramatically lower GFP-positive cells, indicating *RAD54B* knockdown inhibiting HR repair in OVCAR8 cells.

### *RAD54B* knockdown enhanced sensitivity of OvCa cells to olaparib

**Figure 4:**
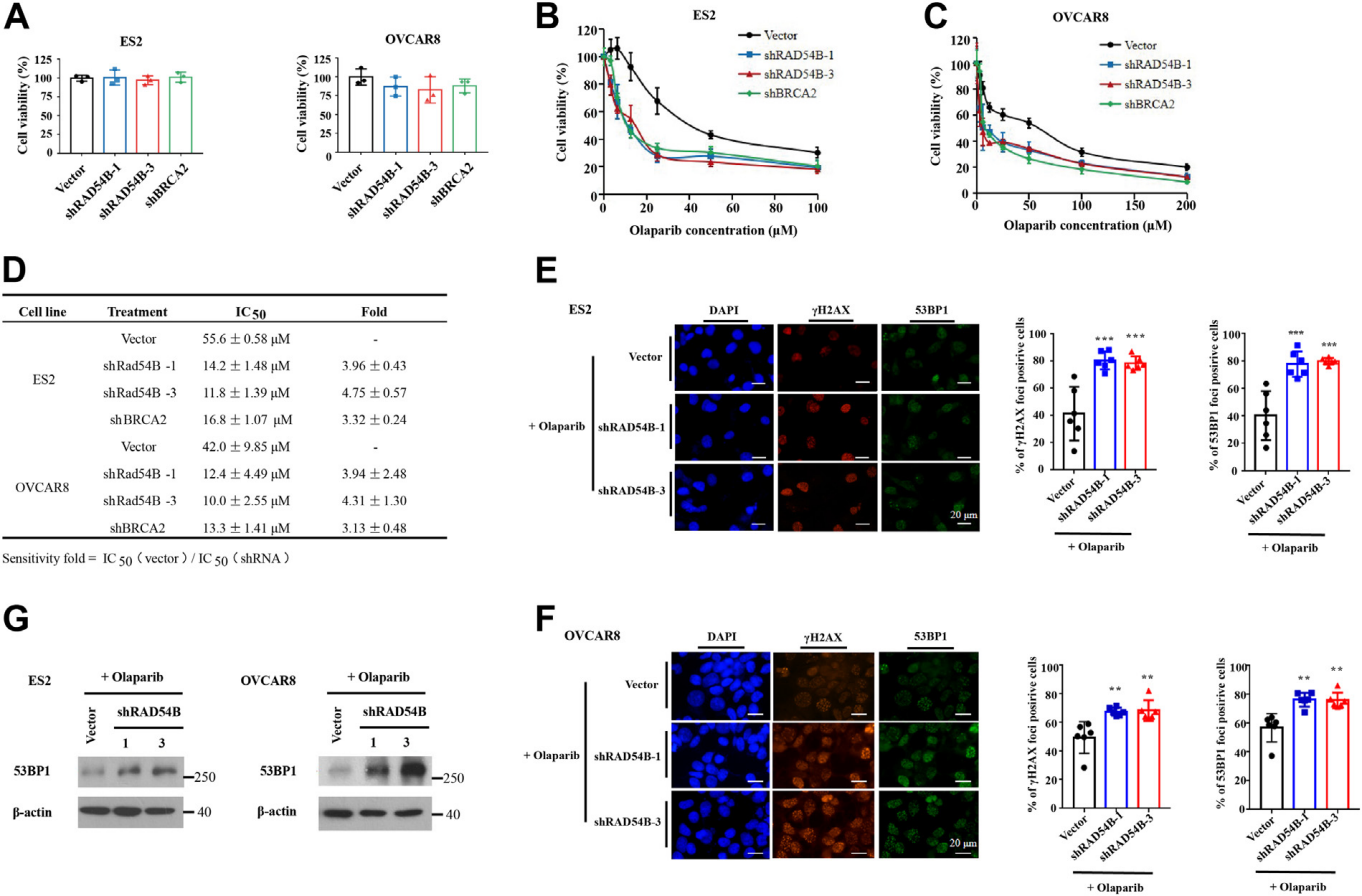
***RAD54B* knockdown enhanced the sensitivities of ovarian cancer cells to olaparib with increased accumulation of DSBs in cells.***A*, *RAD54B* or *BRCA2* knockdown did not significantly affect cell viabilities of ES2 and OVCAR8 cells in the absence of olaparib. *B* and *C*, inhibitory effects of *RAD54B* knockdown on cell viabilities of ES2 and OVCAR8 cells with olaparib treatment at the indicated concentrations. *D*, IC_50_ values of the indicated cell lines with or without *RAD54B* or *BRCA2* knockdown to olaparib treatment. *E* and *F*, representative images and statistics of γH2AX and 53BP1 foci in control or *RAD54B*-knockdowned ES2 (*E*) and OVCAR8 cells (*F*) with olaparib treatment. ES2 and OVCAR8 cells were treated with olaparib at their IC_50_ values (45 and 35 μM, respectively) for 48 h, respectively. The nuclei were stained by DAPI. The positive cells were determined by containing more than five 53BP1 or γH2AX foci. Mean ± SD, n = 3, ∗∗*p* < 0.01, ∗∗∗*p* < 0.001. The scale bar represents 20 μm. *G*, Western blot analyses demonstrating the increased levels of 53BP1 in aforementioned cells. DAPI, 4′,6-diamidino-2-phenylindole; DSB, double-strand break.

**Figure S1:**
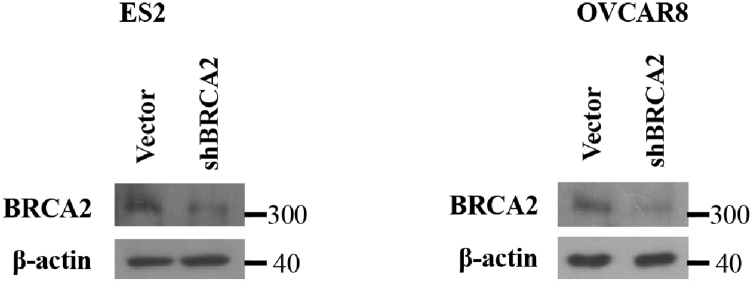


To find out the potential influence of RAD54B malfunctions in OvCa therapy, we tested the effects of *RAD54B* knockdown on the sensitivities of OvCa cells to PARPi treatment. As a positive control, *BRCA2* knockdown was used here (Fig. S1). Before we treated the cells with PARPis, the effects of *RAD54B* or *BRCA2* knockdown on cell viabilities were first tested. The results showed that *RAD54B* knockdown, as well as *BRCA2* knockdown, did not significantly suppress cell viabilities of ES2 and OVCAR8 cells (Fig. 4*A*). We then evaluated the effects of *RAD54B* knockdown on sensitivity of OvCa cells to olaparib. ES2 and OVCAR8 cells with or without *RAD54B* knockdown were treated with olaparib (11) (the first PARPi approved by FDA for the treatment of deleterious germline BRCA-mutated advanced OvCa) at different concentrations. As shown in Figure 4, *B* and *C*, *RAD54B* knockdown significantly increased sensitivities of ES2 and OVCAR8 cells to olaparib. The IC_50_ values were decreased by 3.96-fold and 4.75-fold, respectively, *via* two shRNA constructs in ES2 cells. As for OVCAR8 cells, similar results were obtained (3.94-fold and 4.31-fold, respectively). As the positive control, *BRCA2* knockdown increased the sensitivities of ES2 and OVCAR8 cells to olaparib by 3.32-fold and 3.13-fold, respectively (Fig. 4, *B*–*D*). These results demonstrated that *RAD54B* knockdown significantly enhanced sensitivities of OvCa cells to olaparib.

### *RAD54B* knockdown increased DSBs in OvCa cells after olaparib treatment

It is widely accepted that when cells are treated with PARPis, their single-strand DNA breaks will result in DSBs because they cannot be repaired in time. Cells with sound HR repair machineries might survive in this case, whereas the others might accumulate DSBs and eventually tend to die. Therefore, to investigate whether *RAD54B* knockdown can increase the accumulation of DSBs in olaparib-treated cells, we measured foci formation of γH2AX and 53BP1 in cells (Fig. 4, *E* and *F*). As shown in Figure 4*E*, both γH2AX and 53BP1 foci were significantly increased in *RAD54B*-knockdowned ES2 cells when compared with the vector group after they were treated with olaparib at the same concentration. Similarly, significant increases in percentages of 53BP1 and γH2AX foci–positive cells were also observed in *RAD54B*-knockdowned OVCAR8 cells compared with those of the control groups (Fig. 4*F*). In addition, Western blot results demonstrated increased protein levels of 53BP1 in *RAD54B*-knockdowned ES2 and OVCAR8 cells when they were treated with olaparib (Fig. 4*G*). All these data clearly demonstrated that *RAD54B* knockdown increased DSBs in OvCa cells after olaparib treatment. Considering that the cells of all groups are treated with olaparib under the same conditions, the generation of DSBs should thus be the same. Since the amounts of DSBs depend on the balance between generation and repair, therefore, the accumulation of DSBs implied a compromised HR repair caused by *RAD54B* knockdown.

### *RAD54B* knockdown increased olaparib-induced apoptosis in OvCa cells

**Figure 5:**
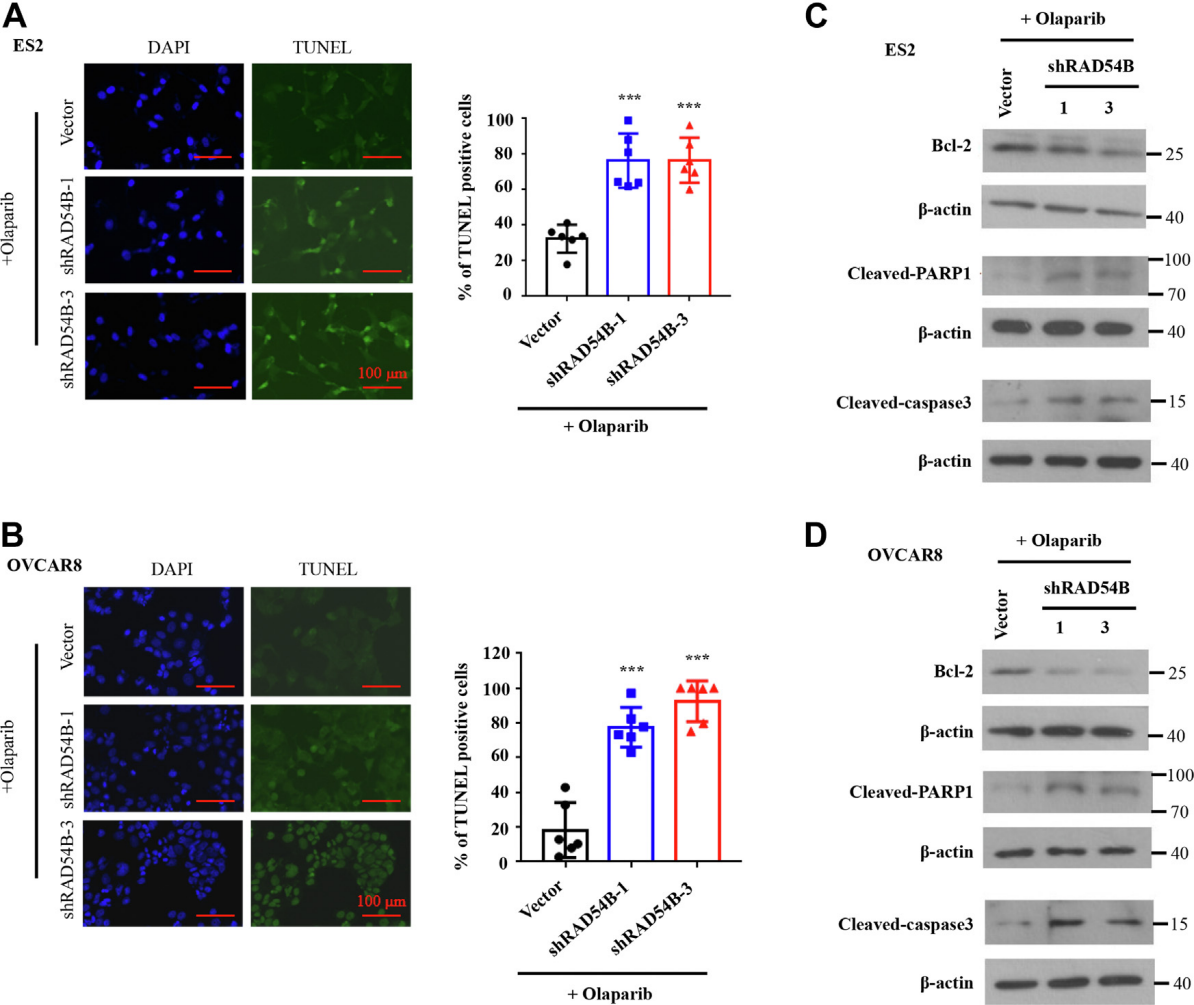
***RAD54B* knockdown enhanced olaparib-induced apoptosis in ES2 and OVCAR8 cells.***A* and *B*, representative images and statistics of TUNEL staining in *RAD54B*-knockdown ES2 (*A*) and OVCAR8 cells (*B*) with olaparib treatment. ES2 and OVCAR8 cells were incubated with 45 and 35 μM of olaparib for 48 h, respectively. Mean ± SD, n = 3. ∗∗∗*p* < 0.001. The scale bar represents 100 μm. *C* and *D*, Western blot for detection of antiapoptotic protein Bcl-2 and proapoptotic proteins cleaved-PARP1 and cleaved-caspase 3 of aforementioned experiments.

Next, we used TUNEL assays to detect whether *RAD54B* knockdown affects apoptosis in drug-treated cells. As shown in Figure 5, *A* and *B*, the results of TUNEL assays demonstrated that *RAD54B* knockdown dramatically increased apoptosis levels in olaparib-treated ES2 and OVCAR8 cells. Consistently, Western blot detection on antiapoptotic protein Bcl-2 and proapoptotic protein cleaved-PARP1 showed that the expression levels of Bcl-2 were decreased in *RAD54B* knockdown groups, whereas the expression levels of cleaved-PARP1 were elevated in olaparib-treated ES2 and OVCAR8 cells (Fig. 5, *C* and *D*). The levels of cleaved-caspase 3, a ubiquitously distributed caspase and a main effector of the apoptotic cascades, were also significantly elevated in *RAD54B*-knockdown ES2 and OVCAR8 cells, when compared with the empty vector groups (Fig. 5, *C* and *D*). These results indicated that *RAD54B* knockdown dramatically increased apoptosis induced by olaparib treatment in OvCa cells.

### *RAD54B* knockdown enhanced the inhibitory effects of olaparib on the growth of ES2 xenograft tumors

**Figure 6:**
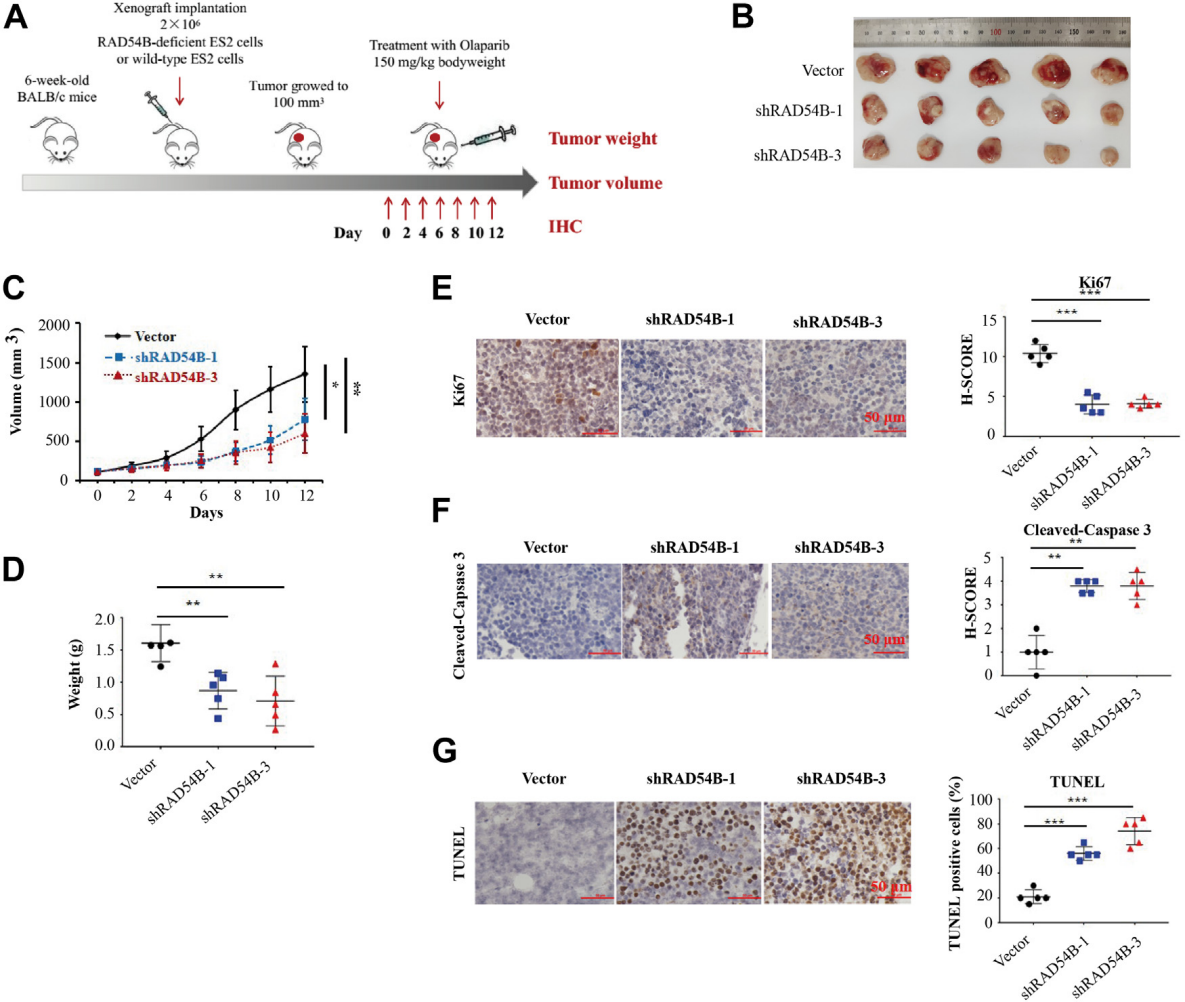
***RAD54B* knockdown enhanced the inhibitory effects of olaparib on the growth of ES2 xenograft tumors.***A*, schematic diagram depicting the ES2 xenograft mouse models. ES2 cells (with or without *RAD54B* knockdown) were subcutaneously injected into the right rib of each immune-deficient nude mice. The xenograft tumors were allowed to grow to 100 mm^3^. Olaparib was dissolved in a solution containing 30% castor seed oil and 4% DMSO before injection. The mice were intraperitoneally injected with olaparib at a dose of 150 mg/kg bodyweight every 2 days and then sacrificed 2 days after six administrations. The xenograft tumors were weighted and measured, and tumor tissues were collected for further studies. *B*, image of tumors after sacrifice. *C*, suppressive effects of *RAD54B* knockdown on the sizes of tumors. Tumor volumes were measured using a caliper. *D*, statistical analyses of the tumor weights. Mean ± SD, n = 5. ∗*p* < 0.05, ∗∗*p* < 0.01. *E*, Ki67 levels were decreased in *RAD54B*-knockdowned ES2 xenograft tumor cells with olaparib treatment. *F*, *RAD54B* knockdown increased the levels of cleaved-caspase 3 in *RAD54B*-knockdowned ES2 xenograft tumor cells with olaparib administration. *G*, effects of *RAD54B* knockdown on apoptosis by counting TUNEL-positive cells. *Left panels*, representative staining images of Ki67, cleaved-caspase 3, and TUNEL determined by IHC. *Right panels*, statistical analyses of the *left panels*. Mean ± SD, n = 3. ∗∗*p* < 0.01, ∗∗∗*p* < 0.001. The scale bar represents 50 μm. DMSO, dimethyl sulfoxide; IHC, immunohistochemistry.

In the next experiments, ES2 cells (with or without *RAD54B* knockdown) were used to generate xenograft tumors (Fig. 6*A*). After olaparib treatment, the results showed that the weight and volumes of tumors in the *RAD54B* knockdown groups were significantly lower, compared with those in the vector group (Fig. 6, *B*–*D*).

To further delineate the molecular mechanisms of the inhibitory effects, the tumor tissues were subsequently analyzed by IHC. Protein levels of Ki67 were first examined in the xenograft tumor tissues. As shown in Figure 6*E*, the Ki67 levels were significantly decreased in *RAD54B*-knockdowned tumor groups. These results suggested that *RAD54B* knockdown significantly inhibited proliferation of OvCa cells with olaparib treatment. We then wondered whether *RAD54B* knockdown promoted apoptosis of olaparib-treated OvCa cells in xenograft tumors. As shown in Figure 6*F*, levels of cleaved-caspase 3 were significantly increased in the *RAD54B*-knockdowned groups. The *RAD54B* knockdown promoted olaparib-induced cell apoptosis in ovarian xenograft tumors. In addition, *in situ* TUNEL assays were carried out to study the DNA fragmentation status in the late stage of apoptosis. In Figure 6*G*, the significant increases of percentages of TUNEL-positive cells in *RAD54B*-knockdowned groups indicated a higher level of apoptosis compared with that in the vector group. No significant difference was observed between the groups of shRAD54B-1 and that of shARD54B-3 on the levels of Ki67, cleaved-caspase 3, and TUNEL (Fig. 6, *E*–*G*). Altogether, these data suggested that *RAD54B* knockdown increased apoptosis and decreased cell proliferation in olaparib-treated ovarian xenograft tumors *in vivo*.

### Expression of wildtype RAD54B rather than RAD54B mutants abolished the effects of RAD54B knockdown

**Figure 7:**
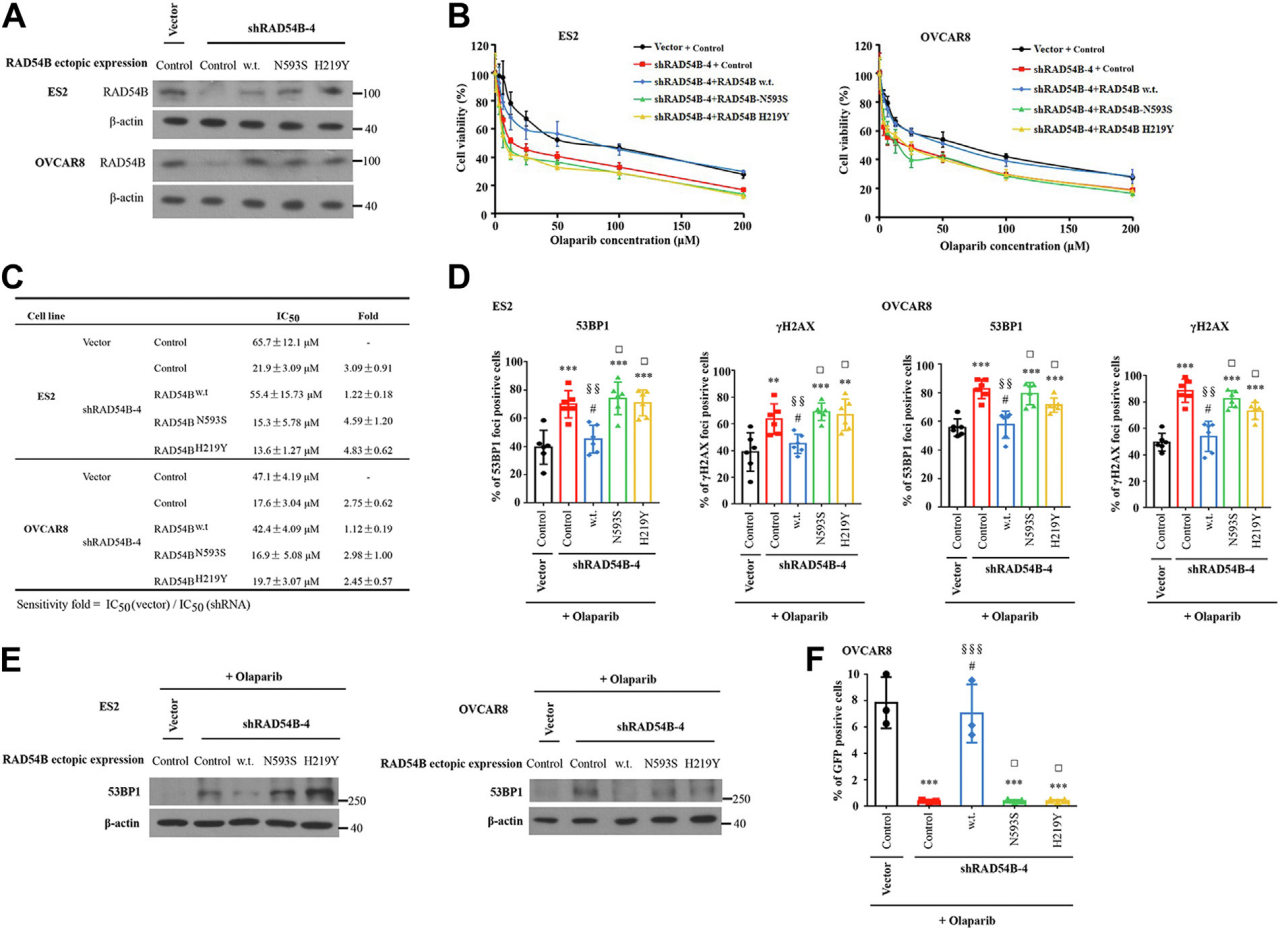
**Restoring wildtype RAD54B rather than RAD54B mutants abolished the effects of *RAD54B* knockdown in ES2 or OVCAR8 cells.***A*, Western blots demonstrated successful knockdown of *RAD54B* with shRAD54B-4 and successful expression of RAD54B^wt^, RAD54B^N593S^. and RAD54B^H219Y^ in *RAD54B*-knockdowned cells. *B*, the cells in *A* were treated with olaparib at the indicated concentrations. *C*, IC_50_ values of the indicated cells to olaparib treatment in *B*. *D*, statistics of 53BP1 and γH2AX foci in cells with the indicated treatments. ES2 and OVCAR8 cells were treated with olaparib at their IC_50_ values (45 and 35 μM, respectively) for 48 h. The nuclei were stained by DAPI. The positive cells were determined by containing more than five 53BP1 or γH2AX foci. Mean ± SD, n = 3. #*p* > 0.05, ∗∗*p* < 0.01, or ∗∗∗*p* < 0.001, compared with the vector-control group; ^□^*p* > 0.05, or ^§§^*p*< 0.01 compared with the shRAD54B-control group. *E*, Western blot analyses demonstrated the levels of 53BP1 in aforementioned cells. *F*, statistics of the percentages of GFP-positive cells in cells with indicated treatments. Mean ± SD, n = 3. #*p* > 0.05 or ∗∗∗*p* < 0.001, compared with the vector-control group; ^□^*p* > 0.05 or ^§§§^*p* < 0.001, compared with the shRAD54B-control group. DAPI, 4′,6-diamidino-2-phenylindole.

**Figure S2:**
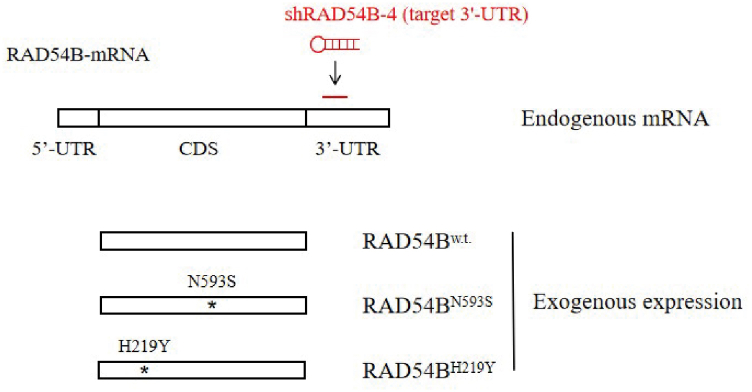


In the aforementioned sections, we knockdowned *RAD54B* to simulate RAD54B malfunctions in cells and achieved the expected results. However, the more important question is whether *RAD54B* mutations we identified in patients indeed impair the normal functions of RAD54B. To answer this question, we conducted the rescue experiments by ectopically expressing different constructs of RAD54B in OvCa cells with *RAD54B* knockdown in advance. Toward this goal, we first knockdowned *RAD54B* in ES2 and OVCAR8 cells by using the shRNAs targeting 3′UTR of *RAD54B* mRNA (Figs. S2 and 7*A*). Subsequently, the *RAD54B*-knockdowned cells were complemented with different variants of RAD54B proteins, namely wildtype, N593S variant, or H219Y variant (Fig. 7*A*). As shown in Figure 7, *B* and *C*, the increased sensitivities of *RAD54B*-knockdowned cells to olaparib were brought back by expression of wildtype RAD54B rather than the two RAD54B mutants in both cell lines.

In addition, we analyzed the foci formation of γH2AX and 53BP1 to investigate whether RAD54B^N593S^ or RAD54B^H219Y^ expression alter the accumulation of DSBs in cells after olaparib treatment (Fig. 7*D*). As shown in Figure 7*D*, both γH2AX and 53BP1 foci were significantly increased in *RAD54B*-knockdowned ES2 and OVCAR8 cells (shRAD54B + control groups) when compared with their corresponding vector + control groups, reproducing the earlier results. At the same time, the expression of wildtype RAD54B significantly decreased the DSBs in *RAD54B*-knockdowned cells when we compared the results of the shRAD54B + wt group to those of shRAD54B + control group (Fig. 7*D*). However, the expression of RAD54B^N593S^ (shRAD54B + RAD54B^N593S^ group) or RAD54B^H219Y^ (shRAD54B + RAD54B^H219Y^ group) did not decrease the DSBs in *RAD54B* knockdown cells, compared with the results from shRAD54B + control groups in either cell line. These results indicate that expression of wildtype RAD54B can restore the functions after *RAD54B* knockdown, but expression of these two RAD54B mutants cannot restore its functions.

Western blot results in addition demonstrated that the increased levels of 53BP1 in *RAD54B*-knockdowned ES2 and OVCAR8 cells treated with olaparib were restored by expression of wildtype RAD54B rather than RAD54B^N593S^ or RAD54B^H219Y^ (Fig. 7*E*). More importantly, the results of DR-GFP assays showed that while expression of wildtype RAD54B restored the HR repair activities in *RAD54B*-knockdowned OVCAR8 cells after olaparib treatment, but both RAD54B^N593S^ and RAD54B^H219Y^ failed to do so (Fig. 7*F*).

Overall, our results demonstrated that expression of wildtype RAD54B rather than RAD54B mutants abolished the effects of *RAD54B* knockdown. These results therefore suggested that RAD54B^N593S^ and RAD54B^H219Y^ probably lost their functions in HR repair.

### Expression of RAD54B in *RAD54B*-mutated tumors

Besides mutations, the expression levels of a protein may also affect its functions. We then measured the expression levels of RAD54B in *RAD54B*-mutated tumors using IHC. As shown in Fig. S3, the expression levels of RAD54B showed no significant differences in *RAD54B*-mutated tumors compared with those in the normal counterparts.

## Discussion

DNA repair has been considered as a critical counteragent in carcinogenesis and potential targets for cancer therapy (59, 60). Proteins implicated in HR repair pathway were frequently mutated in human cancers, such as *BRCA1/2*, *ATM/ATR*, *RAD51*, FA proteins, and others (4, 59, 60). Our WES analyses found that 22 HR genes were mutated in 44 OvCa cases out of a total of 82 cases (Fig. 1*C*). Among them, *RAD54B* has aroused our interest, because the mutation frequency of *RAD54B* was the third highest among 22 mutated HR genes (Fig. 1*B*). Since RAD54B plays an important role in HR repair, its mutation might impair DSB repair in cells. Consistent with this thought, cancer tissues harboring *RAD54B* mutations contain more DSBs (Fig. 2). Considering that PARPis have superior effects in OvCas with HR deficiency (HRD) (61), we hypothesized that OvCa patients with *RAD54B* mutations prefer PARPi treatment. To test this hypothesis, we conducted a series of *in vitro* and *in vivo* experiments. Using DR-GFP assays, we demonstrated that *RAD54B* knockdown inhibited HR repair in OVCAR8 cells (Fig. 3). As expected, OvCa cells with *RAD54B* knockdown exhibited higher sensitivity to olaparib treatment (Fig. 4). Indeed, *RAD54B* knockdown induced accumulation of DSBs (Fig. 4) and aggravated apoptosis of OvCa cells induced by olaparib *in vitro* (Fig. 5). Olaparib inhibited the growth of xenograft tumors of ES2 cells with *RAD54B* knockdown more efficiently (Fig. 6). More importantly, when the endogenous RAD54B was replaced by the RAD54B mutants identified in patients, the OVCAR8 cells remained high sensitivities to olaparib treatment (Fig. 7). All these results suggest that inhibition of RAD54B functions results in defects in HR repair and increases sensitivities to olaparib in OvCa cells.

The earlier studies underestimated the existence of epithelial OvCas with HRD (3, 4, 62, 63, 64, 65, 66). Recent evidence has suggested that approximately 20 to 30% of epithelial OvCas harbor non-BRCA HRD (4). A couple of recent studies identified several frequently mutated HR genes in epithelial OvCas, including *BRCA1/2*, *ATM*, *ATR*, *PTEN*, *RAD51*, *PALB2*, *CHEK2*, *FANCA/M*, and others (63, 64). The mutation frequencies of these HR genes assessed by targeted-next generation sequencing were close to those in our analyses by WES (Fig. 1*B*). However, *RAD54B* was neglected in these studies because of the limited list of the targeted gene panels. Notably, our WES probed into all the mutated HR genes in clinical epithelial OvCa specimens. *RAD54B* mutations were thus identified and aroused our interest. In our WES analyses, 7.3% of OvCa patients harbor *RAD54B* mutations, which is similar to the estimate of 6% in another study by Zhao *et al.* (65).

In the last decade, synthetic lethality led by PARPis in BRCA-deficient cancers has attracted considerable attention. The first PARPi, olaparib, was approved by FDA in maintenance therapy of BRCA1- or BRCA2-deficient OvCa patients in the year of 2014. Great efforts have been made toward the clinical development of PARPis afterward. In view of the superior effects, PARPis were approved for use in the first line and early maintenance therapy along with the new data (67, 68, 69). In addition, scholars and oncologists have recently proposed a concept of HRD scoring for targeted and individualized therapy for OvCa patients (70, 71, 72). PARPi therapies are preferred for patients with higher HRD scores to achieve better outcome (71, 72). HRD scoring is heavily dependent on HRD gene panel, in which *RAD54B* is not currently included. Our study reveals that *RAD54B* is a frequently mutated HR gene in OvCa patients, and its malfunction enhances the sensitivity of cells to olaparib. This evidence supports *RAD54B* as a novel biomarker that should be included in HRD gene panel to benefit more patients.

Overall, our study provides evidence for potential applications of PARPis in synthetic lethal therapy and potential individualized therapy for patients with *RAD54B* mutations.

## Experimental procedures

### Clinical specimens

The study was approved by the Ethics Committee at Jiangsu University. The studies in this work abide by the Declaration of Helsinki principles. The formalin-fixed paraffin-embedded (FFPE) OvCa tissue blocks from 2007 to 2018 were collected at the Affiliated Hospital of Jiangsu University. All the FFPE OvCa and normal tissues were dissected from surgical specimens. Diagnosis was confirmed by pathologists. Major clinical characteristics of these patients are listed in Table S1. In addition, six normal ovarian epithelial tissue samples were collected as negative control.

### WES and variant confirmation

The FFPE OvCa tissue blocks were deparaffinized with xylene. Genomic DNAs were extracted by using QIAamp DNA FFPE Tissue Kit (Qiagen) and randomly fragmented by Corvatis Technology. The selected DNA fragments (distributed between 150 and 250 bp) were dA-tailed. Adapters were ligated to both ends of the DNA fragments. The adapter-ligated DNA fragments were amplified by ligation-mediated PCR, purified, hybridized to human exome array for enrichment, and followed by circularization. The DNA nanoballs were produced by rolling circle amplification. The exome libraries were sequenced by BGISEQ-500 sequencing platform (BGI). Data were processed by BGISEQ-500 basecalling software and stored in FASTQ format.

The FASTQ raw data were filtered and aligned to human reference genome (GRCh37/HG19) using Burrows-Wheeler Aligner software. Variant callings were performed by the GATK-recommended variant analysis process (GATK, https://www.broadinstitute.org/gatk/guide/best-practices). Duplicate reads were removed, and coverage metrics were calculated by Picard-tools (version 1.48; http://picard.sourceforge.net/). Local insertion–deletion realignment and base quality score recalibration were conducted by using GATK. The nonpathogenic variants with mutation frequency >1% in 1000 human genome project database, NHLBI-ESP6500 European American database, and NHLBI-ESP6500 African American database were further removed. The clinical significance and status (including pathogenic/nonpathogenic and tolerated/possible or probably damaging) of variants were identified in COSMIC and ClinVar databases. The potential pathogenic variants were in addition analyzed by using SIFT/PolyPhen2/Mutation assessor/Radial SVM software platforms. See Tables S2 and S3 for a full list of the WES-identified HR mutations.

### IHC

IHC assays were performed as previously described (73, 74, 75). Briefly, the tumor tissues dissected from mice and fixed in neutral-buffered formaldehyde for 3 h at 25 °C. Tissues were dehydrated in 70, 85, 95, and 100% ethanol and incubated in xylene. Subsequently, the tissues were embedded in paraffin, and paraffin-embedded tissues were sliced at 5 μm.

The paraffin-embedded tissue slices were dewaxed followed by antigen retrieval. The slices were then incubated with relevant primary antibodies and incubated with corresponding horseradish peroxidase–conjugated secondary antibodies for 20 min at 25 °C, followed by visualization with 3,3-diaminobenzidine, and then counterstained with hematoxylin. After dehydration and sealing, immunohistochemically stained proteins were observed *in situ* using Nikon Eclipse. Experiments were independently performed three times. The information of antibodies is listed in Table S4.

The evaluations of immunoreactivity were based on the staining extent and intensity by three independent researchers blinded to OvCa sample origins. The staining extent was scored as 5 (81–100%), 4 (61–80%), 3 (41–60%), 2 (21–40%), 1 (1–20%), and 0 (0%). The staining intensity was scored as 5 (extremely strong), 4 (strong), 3 (moderate), 2 (weak), 1 (very weak), and 0 (negative). The sum of the staining extent and intensity was used as the final immunoreactivity score (H-score). In the case of a scoring discrepancy, the slides were re-evaluated by all researchers.

### Cell culture and reagents

OvCa ES2 and OVCAR8 were cultured in RPMI1640, and HO8910 cells were cultured in Dulbecco's modified Eagle's medium, supplemented with 10% fetal bovine serum, and 100 U/ml penicillin and 100 μg/ml streptomycin (Thermo Fisher Scientific) at 37 °C in an atmosphere containing 5% CO_2_, respectively. Olaparib was purchased from Selleck Chemicals and diluted in dimethylsulfoxide. All other chemicals were purchased from Sigma–Aldrich unless otherwise specified.

### Site-directed mutagenesis

Two mutant plasmids pLVX-IRES-Neo containing RAD54B mutant N593S and H219Y were generated by using Mut Express II Fast Mutagenesis Kit V2 (Vazyme Biotech). The primers used in site-directed mutagenesis are listed in Table S5.

### 3-(4,5-Dimethylthiazol-2-yl)-2,5-diphenyltetrazoliumbromide assay

Cell viabilities were measured as previously described (73, 74, 75). Briefly, cells were seeded at 2 × 10^3^ cells per well in 96-well plates and treated with olaparib at different concentrations for 72 h at 37 °C. About 10 μl of 4,5-dimethylthiazol-2-yl)-2,5-diphenyltetrazoliumbromide (Sigma–Aldrich) solution (5 mg/ml) was added in each well and incubated for 4 h at 37 °C. Subsequently, the supernatant in each well was replaced by dimethylsulfoxide. The absorbance was measured by using microplate reader (Bio-Rad) at 550 nm and plotted as cell viability curves using GraphPad Prism (GraphPad Software, Inc). IC_50_ values were calculated by CompuSyn. Error bars correspond to ±SD from three independent experiments.

### Western blotting

Western blotting assays were performed as previously described (73, 74, 75). Briefly, cells were lysed with ice-cold radioimmunoprecipitation assay lysis buffer (50 mM Tris–HCl, pH 7.4, 150 mM NaCl, 1 mM EDTA, 1 mM PMSF, and 1% Triton X-100), and the concentrations of the extracted proteins in supernatant were quantified by bicinchoninic acid protein assay kit (Beyotime Biotech). About 30 μg of each protein sample was loaded on SDS-PAGE gel. After the SDS-PAGE electrophoresis, proteins were transferred on a polyvinylidene difluoride membrane, and blocked with 5% skim milk in Tris-buffered saline with Tween-20 (10 mM Tris–HCl, pH 8.0, 150 mM NaCl, and 0.05% Tween-20). Specific proteins were probed with the relevant primary antibodies followed by secondary antibodies. The immunoblots were visualized with ECL plus Chemiluminescence kit (Thermo Fisher Scientific). The antibodies used are listed in Table S4.

### Lentivirus infections

The shRNAs were purchased from BGI. The sequences are listed in Table S6. Lentivirus was packaged by following the manufacturer’s instructions of a ViraPower Kit from Thermo Fisher Scientific. Production and infection of lentiviruses were conducted as described previously (73). Cells infected with the lentiviruses were screened by 1 μg/ml puromycin.

### HR assays

Shown as in Figure 3*C*, OVCAR-8 cells were transfected with linearized DR-GFP (modified from Addgene #26475) reporter constructs using Etta X-Porator H1 (Etta Biotech) electroporator. G418 was used to select the stable clones. Stable cell lines containing the DR-GFP reporter constructs were infected with lentivirus raised from shRAD54B-1/3 plasmids or vectors and were selected with 1 μg/ml of puromycin. Then, cells were transfected *via* electroporation at a final amount of 1 μg of pCBASceI (Addgene #26477). After 48 h, GFP-positive cells were counted using a Gallios Flow Cytometer (Beckman Coulter). Data were analyzed using FlowJo software (BD Biosciences).

### Immunofluorescence staining

Immunofluorescence staining was carried out as described previously (75, 76). Briefly, cells (3 × 10^4^ cells per well/24-well plate) were fixed in 4% paraformaldehyde for 10 min at 25 °C. Fixed cells were then permeablized with 0.5% Triton X-100 for 5 min at 25 °C and blocked with 3% bovine serum albumin in PBS for 30 min. Next, the cells fixed on a slide were incubated with a selected primary antibody at an appropriate dilution for 2 h at 25 °C in the dark, followed by incubating with an appropriate fluorescent secondary antibody for 1 h at 25 °C. The slides were stained with 1 μg/μl of 4′,6-diamidino-2-phenylindole for 5 min at 25 °C and observed under Nikon Eclipse. The antibodies are described in Table S4.

### TUNEL assays

The paraffin-embedded tissues from mice were fixed in 4% paraformaldehyde solution for 30 min at room temperature. The tissues on slices were then washed with PBS and incubated in 0.5% Triton X-100 for 7 min. The TUNEL assay was carried out by following the manufacturer’s instructions of the TUNEL BrightGreen Apoptosis Detection kit from Vazyme. TUNEL-stained cells were observed *in situ* using Nikon Eclipse.

### *In vivo* xenograft tumor model

All procedures involving mice were approved by the Institutional Animal Care and Use Committee of Jiangsu University. Six-week-old nude BALB/c mice were purchased from Yangzhou University, bred, and maintained in a pathogen-free facility. For xenograft mouse models, 0.2 ml of 1 × 10^7^ of RAD54B-knockdowned ES2 cells was subcutaneously injected into the right rib of each mice. When the tumors grew to about 100 mm^3^, tumor-bearing mice were randomly divided into three groups with five mice per group. The mice were intraperitoneally administrated with olaparib at a dose of 150 mg/kg bodyweight every 2 days. Two days after six administrations, mice were sacrificed, and the xenograft tumors were weighted. Tumor tissues were collected for further studies.

### Statistical analysis

Data are presented as mean ± SD unless otherwise stated. Statistical significance between groups was assessed by Student’s *t* test (two-tailed) or one-way ANOVA using GraphPad Prism, version 7.00. In IHC analyses, *p* values between groups were calculated by Chi-square test (n > 40) or Fisher’s exact test (n < 40). Statistical significance with *p* < 0.05 was considered significant. #*p* ≥ 0.05; ∗*p* < 0.05; ∗∗*p* < 0.01; and ∗∗∗*p* < 0.001.

## Data availability

All data are contained within the article.

## Ethics

The study was approved by the Ethics Committee at Jiangsu University. The studies in this work abide by the Declaration of Helsinki principles. The animal studies were reviewed and approved by Jiangsu University Institutional Animal Care and Use Committee.

## Supporting information

This article contains supporting information.

## Conflict of interest

The authors declare that they have no conflicts of interest with the contents of this article.

## Figures and Tables

**Figure S3:**