# Supplementary material for: RAD54B mutations enhance the sensitivity of ovarian cancer cells to poly(ADP-ribose) polymerase (PARP) inhibitors
Source: J Biol Chem. 2022 Aug 9;298(9):102354. doi: 10.1016/j.jbc.2022.102354 (PMC9463535; doi:10.1016/j.jbc.2022.102354)
Supplement: Table S1 [file mmc1.docx]

**Table S1. Clinical and pathological characteristics of patients with ovarian cancers in the current study.**

| **Clinicopathological characteristics** | **Age** | |
| --- | --- | --- |
| **Age** | | |
| Median | 54 | |
| Range | 23-83 | |
| **Clinicopathological characteristics** | **Number of patients** | |
|  | **Number (n)** | **% of total (n_total_=82)** |
| **Age** |  |  |
| < 40 | 7 | 8.5 |
| 40 to 60 | 55 | 67.1 |
| > 60 | 20 | 24.4 |
| **Histology** | | |
| Serous ovarain carcinoma | 45 | 54.9 |
| Mucinous ovarian carcinomas | 16 | 19.5 |
| Ovarian clear-cell carcinomas | 12 | 14.6 |
| Unclassified (others) | 9 | 11.0 |
| **FIGO stage** | | |
| I | 23 | 28.0 |
| II | 13 | 15.9 |
| III | 33 | 40.2 |
| IV | 12 | 14.6 |
| Unclassified | 1 | 1.2 |
| **Differentiation** | | |
| Low | 39 | 47.6 |
| Middle | 9 | 11.0 |
| High | 34 | 41.5 |
| **Metastasis** | | |
| Lymph node metastasis | 34 | 41.5 |
| Developed peritoneal metastasis | 34 | 41.5 |
| Non metastasis | 14 | 17.1 |
