# Supplementary material for: RAD54B mutations enhance the sensitivity of ovarian cancer cells to poly(ADP-ribose) polymerase (PARP) inhibitors
Source: J Biol Chem. 2022 Aug 9;298(9):102354. doi: 10.1016/j.jbc.2022.102354 (PMC9463535; doi:10.1016/j.jbc.2022.102354)
Supplement: Table S2 [file mmc2.docx]

**Table S2. The missense, framshift, stop gained, in-fram deletion and start lost variants of HR-related genes.**

| **Patients** | **Genes** | **Mutations** | **COSMIC** | **ClinVar** | **SIFT** | **PolyPhen2** | **Mutation**  **Assessor** | **Radial**  **SVM** |
| --- | --- | --- | --- | --- | --- | --- | --- | --- |
| 1. **Missense variants** | | | | | | | | |
| N°15 mucinous | ATM | NM_000051.3:p.Ser2168Leu/c.6503C>T | Pathogenic | Uncertain significance | Damaging | Possibly damaging | Medium | Tolerated |
| N°54 serous | ATR | NM_001184.3:p.Arg2217His/c.6650G>A | / | / | Damaging | Possibly damaging | Medium | Tolerated |
| N°62 serous | ATR | NM_001184.3:p.Arg109Trp/c.325C>T | / | Uncertain significance | Damaging | Possibly damaging | Neutral | Tolerated |
| N°77 serous | ATR | NM_001184.3:p.Leu213Val/c.637C>G | / | / | Damaging | Benign | Neutral | Tolerated |
| N°80 serous | ATR | NM_001184.3:p.Ala669Pro/c.2005G>C | / | / | Damaging | Benign | Neutral | Tolerated |
| N°83 serous | BLM | NM_000057.3:p.Lys812Gln/c.2434A>C | / | / | Damaging | Possibly damaging | Low | Tolerated |
| N°48 serous | BRCA1 | NM_007300.3:p.Pro1150Ser/c.3448C>T | / | Uncertain significance | Damaging | Possibly damaging | Medium | Damaging |
| N°53 clear-cell | BRCA1 | NM_007300.3:p.Gly275Asp/c.824G>A | / | Uncertain significance | Damaging | Possibly damaging | High | Damaging |
| N°3 clear-cell | BRCA2 | NM_000059.3:p.Glu2981Lys/c.8941G>A | Pathogenic | Pathogenic | Damaging | Benign | Medium | Tolerated |
| N°20 serous | BRCA2 | NM_000059.3:p.Ile1436Thr/c.4307T>C | / | / | Tolerated | Possibly damaging | Neutral | Tolerated |
| N°26 serous | BRCA2 | NM_000059.3:p.Gly2508Ser/c.7522G>A | / | Pathogenic | Damaging | Probably damaging | Medium | Damaging |
| N°48 serous  N°65 serous | BRIP1 | NM_032043.2:p.Arg814Cys/c.2440C>T | / | Conflicting | Damaging | Probably damaging | Medium | Damaging |
| N°82 serous | BRIP1 | NM_032043.2:p.Ile32Phe/c.94A>T | / | / | Damaging | Probably damaging | Medium | Damaging |
| N°3 clear-cell | CHEK2 | NM_001005735.1:p.Ala134Val/c.401C>T | / | / | Tolerated | Possibly damaging | / | Tolerated |
| N°39 mucinous | DNA2 | NM_001080449.2:p.Arg311Cys/c.931C>T | / | / | Damaging | Probably damaging | High | Damaging |
| N°20 serous | FANCA | NM_000135.2:p.Ser119Cys/c.356C>G | / | / | Damaging | Benign | Medium | Tolerated |
| N°82 serous | FANCA | NM_000135.2:p.Arg591Gln/c.1772G>A | / | Uncertain significance | Damaging | Benign | Medium | Damaging |
| N°84 serous | FANCA | NM_000135.2:p.Cys1159Ser/c.3476G>C | / | Uncertain significance | Damaging | Possibly damaging | Medium | Damaging |
| N°36 serous | FANCC | NM_000136.2:p.Arg334Trp/c.1000C>T | / | / | Damaging | Possibly damaging | Neutral | Tolerated |
| N°83 serous | FANCD2 | NM_033084.4:p.Leu436Met/c.1306C>A | Pathogenic | / | Damaging | Probably damaging | Medium | Tolerated |
| N°21 serous | FANCE | NM_021922.2:p.Ala530Pro/c.1588G>C | / | / | Damaging | Probably damaging | Medium | Tolerated |
| N°40 serous | *FANCF* | NM_022725.3:p.Trp26Arg/c.76T>A | / | / | Damaging | Probably damaging | Medium | Damaging |
| N°3 clear-cell | *FANCG* | NM_004629.1:p.Pro590Ala/c.1768C>G | / | / | Damaging | Probably damaging | Medium | Damaging |
| N°14 serous | *FANCG* | NM_004629.1:p.Pro385Ser/c.1153C>T | / | / | Tolerated | Possibly damaging | Medium | Tolerated |
| N°30 serous | *FANCI* | NM_001113378.1:p.Met363Thr/c.1088T>C | / | / | Tolerated | Possibly damaging | Medium | Tolerated |
| N°39 mucinous | *FANCI* | NM_001113378.1:p.Leu95Gln/c.284T>A | / | / | Damaging | Benign | Neutral | Tolerated |
| N°3 clear-cell | *FANCM* | NM_020937.2:p.Gly546Asp/c.1637G>A | / | / | Damaging | Probably damaging | Medium | Damaging |
| N°19 serous | *FANCM* | NM_020937.2:p.Ser1713Phe/c.5138C>T | / | / | Damaging | Benign | Medium | Tolerated |
| N°54 serous | *FANCM* | NM_020937.3:p.Pro1595His/c.4784C>A | / | Uncertain significance | Damaging | Probably damaging | Medium | Damaging |
| N°77 serous | *FANCM* | NM_020937.3:p.Ser1788Cys/c.5363C>G | / | / | Damaging | Possibly damaging | Medium | Tolerated |
| N°79 serous | *FANCM* | NM_020937.3:p.Ser776Arg/c.2328C>G | / | / | Damaging | Benign | Neutral | Tolerated |
| N°24 serous  N°53 clear-cell | *PALB2* | NM_024675.3:p.Glu352Gln/c.1054G>C | / | Uncertain significance | Tolerated | Possibly damaging | Medium | Tolerated |
| N°48 serous | *PALB2* | NM_024675.3:p.Leu901Pro/c.2702T>C | / | / | Tolerated | Probably damaging | Medium | Tolerated |
| N°67 serous | *PALB2* | NM_024675.3:p.Pro405Ala/c.1213C>G | / | Uncertain significance | Damaging | Probably damaging | Medium | Tolerated |
| N°78 serous | *PALB2* | NM_024675.3:p.Glu1018Asp/c.3054G>C | / | Conflicting | Damaging | Probably damaging | Medium | Tolerated |
| N°19 serous | *RAD51B* | NM_133509.3:p.Thr107Lys/c.320C>A | / | / | Damaging | Possibly damaging | Medium | Tolerated |
| N°42 serous | *RAD51C* | NM_058216.2:p.Gly306Val/c.917G>T | / | / | Damaging | Probably damaging | Medium | Tolerated |
| N°14 serous  N°15 serous  N°76 serous | *Rad54B* | NM_012415.3:p.Asn593Ser/c.1778A>G | / | Pathogenic | Damaging | Probably damaging | Medium | Damaging |
| N°21 serous | *Rad54B* | NM_012415.3:p.His219Tyr/c.655C>T | / | / | Damaging | Benign | Neutral | Tolerated |
| N°77 serous | *Rad54B* | NM_012415.3:p.Tyr738His/c.2212T>C | / | / | Damaging | Probably damaging | Medium | Damaging |
| N°3 clear-cell | *RAD54L* | NM_001142548.1:p.Pro597Ser/c.1789C>T | / | / | Damaging | Probably damaging | High | Damaging |
| N°82 serous | *RAD54L* | NM_001142548.1:p.Ile583Thr/c.1748T>C | / | / | Damaging | Possibly damaging | Neutral | Tolerated |
| N°63clear-cell | *RPA1* | NM_002945.3:p.Val286Ile/c.856G>A | Pathogenic |  |  | Benign | Neutral | Tolerated |
| 1. **Framshift variants** | | | | | | | | |
| N°3 clear-cell | *ATM* | NM_000051.3:p.Tyr2149_Ala2150fs/c.6445_6446insTCTGGCC | / | / | / | / | / | / |
| N°34 serous | *ATM* | NM_000051.3:p.Ser214fs/c.640dupT | / | / | / | / | / | / |
| N°50 clear-cell | *ATM* | NM_000051.3:p.Gly1130fs/c.3388_3391delGGAA | / | / | / | / | / | / |
| N°3 clear-cell | *BLM* | NM_000057.3:p.Val1180_Leu1181fs/c.3539_3540insAACGTTGC | / | / | / | / | / | / |
| N°64 serous | *BRCA1* | NM_007300.3:p.Ile1845fs/c.5533_5540delATTGGGCA | / | Pathogenic | / | / | / | / |
| N°70 serous | *BRCA1* | NM_007300.3:p.Lys38fs/c.112_113delAA | / | Pathogenic | / | / | / | / |
| N°80 serous | *BRCA1* | NM_007300.3:p.Pro1512fs/c.4535delC | / | / | / | / | / | / |
| N°11 serous | *BRCA2* | NM_000059.3:p.Ser142_Pro143fs/c.424_425insTGAG | / | / | / | / | / | / |
| N°12 mucinous | *BRCA2* | NM_000059.3:p.Lys936fs/c.2806_2809delAAAC | / | Pathogenic | / | / | / | / |
| N°14 serous | *BRCA2* | NM_000059.3:p.Tyr1655fs/c.4965delC | / | Pathogenic | / | / | / | / |
| N°38 serous | *BRCA2* | NM_000059.3:p.Asn588fs/c.1764delT | / | / | / | / | / | / |
| N°73 serous | *BRCA2* | NM_000059.3:p.Asn1805fs/c.5412_5413insTAAG | / | / | / | / | / | / |
| N°77 serous | *BRCA2* | NM_000059.3:p.Ser205fs/c.610delC | / | Pathogenic | / | / | / | / |
| N°34 serous | *DNA2* | NM_001080449.2:p.Ser779fs/c.2335delT | / | / | / | / | / | / |
| N°3 clear-cell | *FANCD2* | NM_033084.3:p.Ser1145_Thr1146fs/c.3433_3434insG | / | / | / | / | / | / |
| N°3 clear-cell | *RAD51B* | NM_133509.3:p.Thr353_Gln354fs/c.1058_1059insC | / | / | / | / | / | / |
| N°34 serous | *RAD51B* | NM_001321821.1:p.Arg231fs/c.692_695delGAAA | / | / | / | / | / | / |
| N°39 mucinous | *RAD51B* | NM_133509.3:p.Pro369fs/c.1104_1105dupTC | / | / | / | / | / | / |
| N°39 mucinous | *RAD51B* | NM_133509.3:p.Glu370fs/c.1107_1108delTG | / | / | / | / | / | / |
| N°34 serous | *RAD51C* | NM_058216.2:p.Gly113fs/c.338delG | / | / | / | / | / | / |
| N°3 clear-cell | *RAD54L* | NM_001142548.1:p.Leu10fs/c.30delG | / | / | / | / | / | / |
| N°84 serous | *RAD54L* | NM_001142548.1:p.Asp183fs/c.537_547dupCATCATGGCTG | / | / | / | / | / | / |
| 1. **Stop gained variants** | | | | | | | | |
| N°82 serous | *ATM* | NM_000051.3:p.Gln1331*/c.3991C>T | / | / | / | / | / | / |
| N°47 serous | *ATR* | NM_001184.3:p.Tyr1773*/c.5319C>G | / | / | / | / | / | / |
| N°23 serous  N°36 serous | *BRCA1* | NM_007300.3:p.Gln1546*/c.4636C>T | / | / | / | / | / | / |
| N°42 serous | *BRCA1* | NM_007300.3:p.Glu720*/c.2158G>T | / | Pathogenic | / | / | / | / |
| N°57 mucinous | *BRCA2* | NM_000059.3:p.Gln1509*/c.4525C>T | / | Pathogenic | / | / | / | / |
| N°74 serous  N°77 serous | *BRCA2* | NM_000059.3:p.Lys1394*/c.4180A>T | / | / | / | / | / | / |
| N°3 clear-cell | *FANCA* | NM_000135.2:p.Glu886*/c.2656G>T | / | / | / | / | / | / |
| N°58 mucinous | *FANCM* | NM_020937.3:p.Gln1885*/c.5653C>T | / | / | / | / | / | / |
| N°28 serous  N°39 mucinous | *RAD51B* | NM_133509.3:p.Gln371*/c.1111C>T | / | / | / | / | / | / |
| N°58 mucinous | *Rad54B* | NM_012415.3:p.Gln209*/c.625C>T | / | / | / | / | / | / |
| 1. **In-frame deletion variants** | | | | | | | | |
| N°81 serous | *BRIP1* | NM_032043.2:p.Val720del/c.2158_2160delGTG | / | Uncertain significance | / | / | / | / |
| N°61 serous | *FANCD2* | NM_033084.3:p.Asp1410_Ser1412del/c.4230_4235delTGAGAG | / | / | / | / | / | / |
| 1. **Start lost variant** | | | | | | | | |
| N°3 celar-cell | *FANCD2* | NM_033084.3:p.Met1?/c.3G>A | / | / | / | / | / | / |
