# Supplementary material for: RAD54B mutations enhance the sensitivity of ovarian cancer cells to poly(ADP-ribose) polymerase (PARP) inhibitors
Source: J Biol Chem. 2022 Aug 9;298(9):102354. doi: 10.1016/j.jbc.2022.102354 (PMC9463535; doi:10.1016/j.jbc.2022.102354)
Supplement: Table S4 [file mmc4.docx]

**Table S4. Antibodies used in this study.**

| **Primary antidodies** | **Source** | **Company** | **Application** | **Dilution ratio** |
| --- | --- | --- | --- | --- |
| RAD54B | Mouse | Santa | Western blot | 1:1000 |
|  |  |  | Immunohistochemistry | 1:300 |
| β-actin | Mouse | Cell Signaling Technology | Western blot | 1:1000 |
| 53BP1 | Rabbit | BETHYL | Western blot | 1:1500 |
|  |  |  | Immunofluorescence | 1:1000 |
| γH2AX | Rabbit | Cell Signaling Technology | Immunofluorescence | 1:1000 |
| Ki67 | Rabbit | CusAbio | Immunohistochemistry | 1:100 |
| Cleaved-Caspase3 (RLC006) | Rabbit | Ruiyingbio | Immunohistochemistry | 1:300 |
| Bcl-2 | Mouse | CusAbio | Western blot | 1:1500 |
| PARP1 | Rabbit | Cell Signaling Technology | Western blot | 1:1200 |
| BRCA2 | Rabbit | Abcam | Western blot | 1:1000 |
| **Secondary antibodies** | **Source** | **Company** | **Application** | **Dilution ratio** |
| Goat anti-mouse Alexa-fluor 594 | Goat | Life Technologies | Immunofluorescence | 1:1000 |
| Goat anti-rabbit Alexa-fluor 488 | Goat | Life Technologies | Immunofluorescence | 1:1000 |
| Goat anti-Mouse HRP | Goat | Jackson ImmunoResearch | Western blot | 1:5000 |
|  |  |  | Immunohistochemistry | 1:1000 |
| Goat anti-Rabbit HRP | Goat | Jackson ImmunoResearch | Western blot | 1:5000 |
|  |  |  | Immunohistochemistry | 1:1000 |
