# Supplementary material for: RAD54B mutations enhance the sensitivity of ovarian cancer cells to poly(ADP-ribose) polymerase (PARP) inhibitors
Source: J Biol Chem. 2022 Aug 9;298(9):102354. doi: 10.1016/j.jbc.2022.102354 (PMC9463535; doi:10.1016/j.jbc.2022.102354)
Supplement: Table S5 [file mmc5.docx]

**Table S5. Primers used for site-directed mutagenesis in this study.**

| **Name** | **Sequences (5’ to 3’)** |
| --- | --- |
| RAD54B^N593S^ | 5'- ACTGTGCAGTCACCCCTGCCTTTTGTTCAACT -3'  5'- AGGGGTGACTGCACAGTTTTTTAAGAGCTCCTATAC -3' |
| RAD54B^H219Y^ | 5'- GCTATCTCGTATTCTTCTCAGGTTGCCAGGAA -3'  5'- GAAGAATACGAGATAGCAGTACTTCCTCCTCC -3' |
