# Supplementary material for: RAD54B mutations enhance the sensitivity of ovarian cancer cells to poly(ADP-ribose) polymerase (PARP) inhibitors
Source: J Biol Chem. 2022 Aug 9;298(9):102354. doi: 10.1016/j.jbc.2022.102354 (PMC9463535; doi:10.1016/j.jbc.2022.102354)
Supplement: Table S6 [file mmc6.docx]

**Table S6. shRNA sequences used in this study.**

| **Name** | **Sense Sequences (5’ to 3’)** | **Antisense Sequences (5’ to 3’)** |
| --- | --- | --- |
| shRAD54B-1 | 5’-CCGGGCAGATTGTTGATGGCTTTAACTCGAGTTAAAGCCATCAACAATCTGCTTTTTG-3’ | 5’-CAAAAAGCAGATTGTTGATGGCTTTAACTCGAGTTAAAGCCATCAACAATCTGCCCGG-3’ |
| shRAD54B-3 | 5’-CCGGGACATTCCATTGCTCTTCTTTCTCGAGAAAGAAGAGCAATGGAATGTCTTTTTG-3’ | 5’-CAAAAAGACATTCCATTGCTCTTCTTTCTCGAGAAAGAAGAGCAATGGAATGTCCCGG-3’ |
| shRAD54B-4 (targeting 3’UTR) | 5’-CACCGAAAGATTACTTCTGACATTCCGAAGAATGTCAGAAGTAATCTTTC-3’ | 5’-AAAAGAAAGATTACTTCTGACATTCTTCGGAATGTCAGAAGTAATCTTTC-3’ |
| shBRCA2 | 5’-CCGGGCAGCCATTAAATTGTCCATACTCGAGTATGGACAATTTAATGGCTGCTTTTTG-3’ | 5’-AATTCAAAAAGCAGCCATTAAATTGTCCATACTCGAGTATGGACAATTTAATGGCTGC-3’ |
