# Supplementary material for: RAD54B mutations enhance the sensitivity of ovarian cancer cells to poly(ADP-ribose) polymerase (PARP) inhibitors
Source: J Biol Chem. 2022 Aug 9;298(9):102354. doi: 10.1016/j.jbc.2022.102354 (PMC9463535; doi:10.1016/j.jbc.2022.102354)
Supplement: Supplementary Figure Legends [file mmc7.doc]

**Figure S1. BRCA2 knockdown in ES2 and OVCAR8 cells.**

**Figure S2. Schematic diagram of RAD54B-knockdown by shRAD54B-4 targeting 3’UTR of human RAD54B-mRNA, transcript variant 1 (NCBI NM_012415.3).**

**Figure S3. No significant differences of RAD54B levels in RAD54B-mutated ovarian cancer tissues versus normal ovarian epithelial tissues.** *A*, Representative images of IHC showing RAD54B staining in ovarian cancer tissues from the 5 patients harboring RAD54B mutations (N°14, 15, 76, 21 and 77) and in normal ovarian epithelial tissues. Of note, the RAD54B mutation in N°58 specimen is a short truncated form of RAD54B protein (stop at G209) which does not contain the epitope (amino acid 801-900) recognized by our RAD54B antibody (Santa cruz, sc-101234). Therefore, N°58 specimen was excluded from the IHC experiments. Scale bar = 50 µm. *B*, Statistical analyses of A. Mean ± SD, n = 5. # p >0.05, no significant difference.
